# A first report of hydroxylated apatite as structural biomineral in Loasaceae – plants’ teeth against herbivores

**DOI:** 10.1038/srep26073

**Published:** 2016-05-19

**Authors:** Hans-Jürgen Ensikat, Thorsten Geisler, Maximilian Weigend

**Affiliations:** 1 Institut für Biodiversität der Pflanzen, Universität Bonn, Meckenheimer Allee 170, 53115 Bonn, Germany; 2 Steinmann-Institut für Geologie, Mineralogie und Paläontologie, Universität Bonn, Poppelsdorfer Schloss, 53115 Bonn, Germany

## Abstract

Biomineralization provides living organisms with various materials for the formation of resilient structures. Calcium phosphate is the main component of teeth and bones in vertebrates, whereas especially silica serves for the protection against herbivores on many plant surfaces. Functional calcium phosphate structures are well-known from the animal kingdom, but had not so far been reported from higher plants. Here, we document the occurrence of calcium phosphate biomineralization in the South-American plant group Loasaceae (rock nettle family), which have stinging trichomes similar to those of the well-known stinging nettles (*Urtica*). Stinging hairs and the smaller, glochidiate trichomes contained nanocrystalline hydroxylated apatite, especially in their distal portions, replacing the silica found in analogous structures of other flowering plants. This could be demonstrated by chemical, spectroscopic, and diffraction analyses. Some species of Loasaceae contained both calcium phosphate and silica in addition to calcium carbonate. The intriguing discovery of structural hydroxylated apatite in plants invites further studies, e.g., on its systematic distribution across the family, the genetic and cellular control of plant biomineralization, the properties and ultrastructure of calcium phosphate. It may prove the starting point for the development of biomimetic calcium phosphate composites based on a cellulose matrix.

Loasaceae, a nearly exclusively New World plant family, are often equipped with extremely effective stinging hairs and bear a dense cover of hooked or barbed (“glochidiate”), mineralized trichomes with fascinating shapes. The phytochemistry of Loasaceae is relatively well-studied^1^,^2^, but very little is known about their biomineralization. Biomineralization as such is a fairly common phenomenon in plants^3^ and has been known for over 150 years^4^, with phytoliths, cystoliths and mineralized trichomes the most widespread phenomena^5^,^3^,^6^. Calcium phosphates play the leading role as biominerals in animals, with hydrated-hydroxylated as well as carbonated apatite, Ca_10−x_[(PO_4_)_6−x_(CO_3_)_x_](OH)_2−x_·*n*H_2_O, where *n* ~ 1.5, as the primary mineral component in teeth and bones^7^,^8^, whereas in higher plants the range of biominerals seemed to be limited to silica, calcium carbonate and calcium oxalate^3^,^6^,^12^. There is only a single reference to the possible occurrence of carbonated and hydroxylated apatite, sometimes also referred to as dahllite, in tracheophytes^6^ and a passing note on the possible presence of intracellular calcium phosphate crystals in *Capsicum*^13^, but no evidence for structural phosphate biominerals in plants. Conversely, both silica (amorphous SiO_2_) and calcium carbonate (CaCO_3_) have been shown to play important roles on the plant surface, especially in mineralized plant trichomes such as the stinging hairs of *Urtica*^14^,^15^ and the glochidia of Boraginaceae^16^. Mineralized trichomes have their primary function in herbivore deterrence, but may also be important for diaspore protection and dispersal^15^,^16^,^17^,^18^,^19^.

Several plant families are characterized by a particularly complex hair cover, and the stinging hairs found in a few plant families are amongst the most striking plant microstructures. Their peculiar function as a hypodermic syringe, injecting toxins into animals coming into contact with them, requires a particular mechanical strength which cannot be easily accommodated by purely cellulose-based structures. The stinging hairs of nettles (*Urtica*, Urticaceae) have long been known to be mineralized with calcium carbonate and silica^4^, occurring especially in the apical portion^14^,^20^. Conversely, the elementary composition of the stinging hairs found in other plant families is poorly known. The stinging hairs of Loasaceae are morphologically very similar to those of Urticaceae, with a pluricellular pedestal at the base and a long unicellular stinging hair at the top, having a bulbous base and long, needle-shaped apex, terminating in a small bulbous appendage. This apical bulb becomes easily detached upon mechanical stress, freeing the sharp tip of the trichome and thus opening into the lumen of the cell like a hypodermic syringe, ejecting the irritant cell content^14^. Loasaceae are usually also provided with much shorter, unicellular trichomes with a rough or barbed surface, the so-called scabrid or glochidiate trichomes^21^.

Calcium phosphate, the main inorganic component in bones and teeth of animals, has not been known to contribute to any structural components of plant bodies, although the presence of phosphorus in plant trichomes together with calcium and other metal cations has been reported in studies on metal accumulation in plants^22^,^23^. In line with these findings, we observed phosphorus in minor concentrations on the mineralized cell walls of *Urtica* stinging hairs (see Supplementary Fig. 1a) and several other plant trichomes.

In the present study, we report the intriguing and unambiguous discovery of nano-crystalline hydroxylated apatite in the complex trichome cover of Loasaceae, this being the first report of calcium phosphate playing a role as a structural biomineral in plants.

## Results

**Figure 1:**
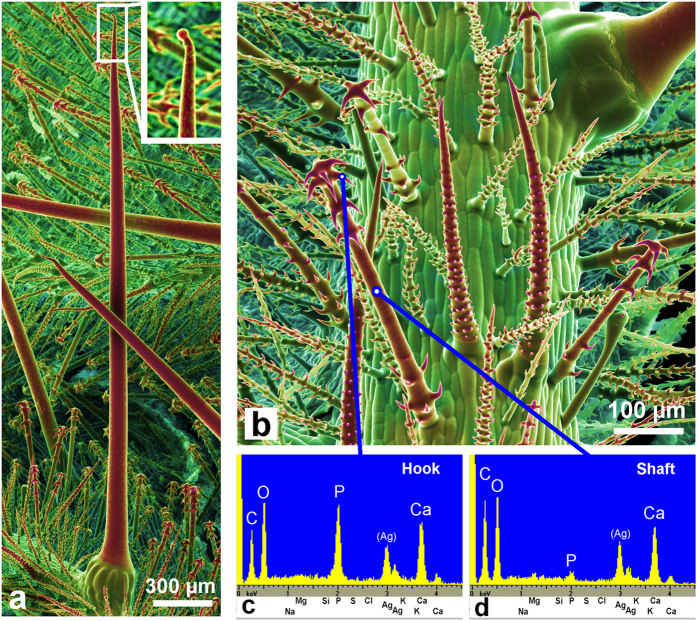
Cryo-SEM images of mineralized trichomes on a *Loasa pallida* leaf. (**a**) The stinging hairs resemble those of *Urtica* (stinging nettle) with a small bulb at the apex (magnified inset). (**b**) Small trichomes with sharp hooks cover the whole surface of the plant. The color images are dual-detector false-color images combining topographical and compositional contrast. A color shift towards red indicates a higher content of calcium and phosphorus (Ca, P). (**c**,**d**) EDX element analyses show high phosphorus and calcium concentrations in the trichome hooks, and high calcium, but low phosphorus content in the trichome shaft, indicating a calcium carbonate phase. The other epidermal cells are not mineralized. The small silver (Ag) peaks originate from a thin metal coating of the sample.

**Figure 2:**
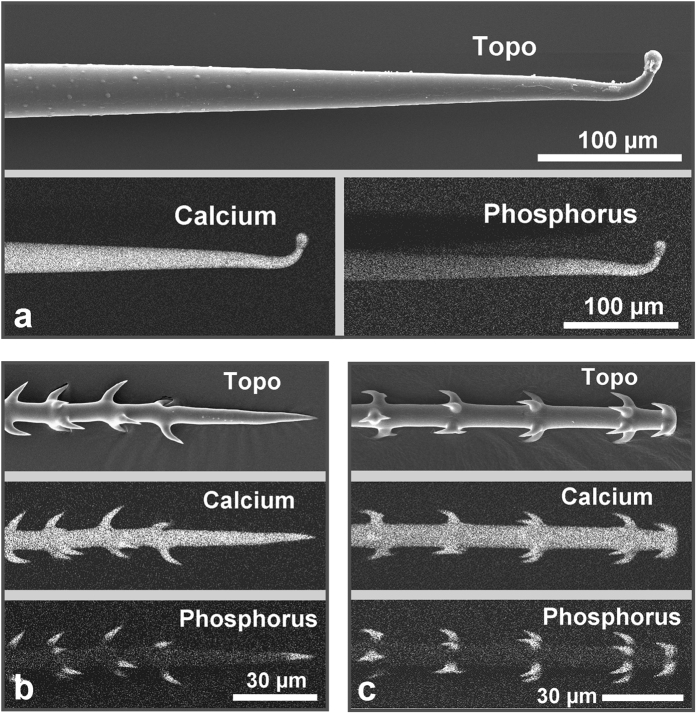
Localization of the mineral elements calcium and phosphorus in the trichomes. SE-image (topography) and element mapping for Ca and P of a stinging hair tip (**a**) and of two types of glochidiate hairs (**b**,**c**). The whole hairs contain Ca, whereas P occurs only in the apical region of the stinging hair and in the hooks and tips of the glochids.

**Figure 3:**
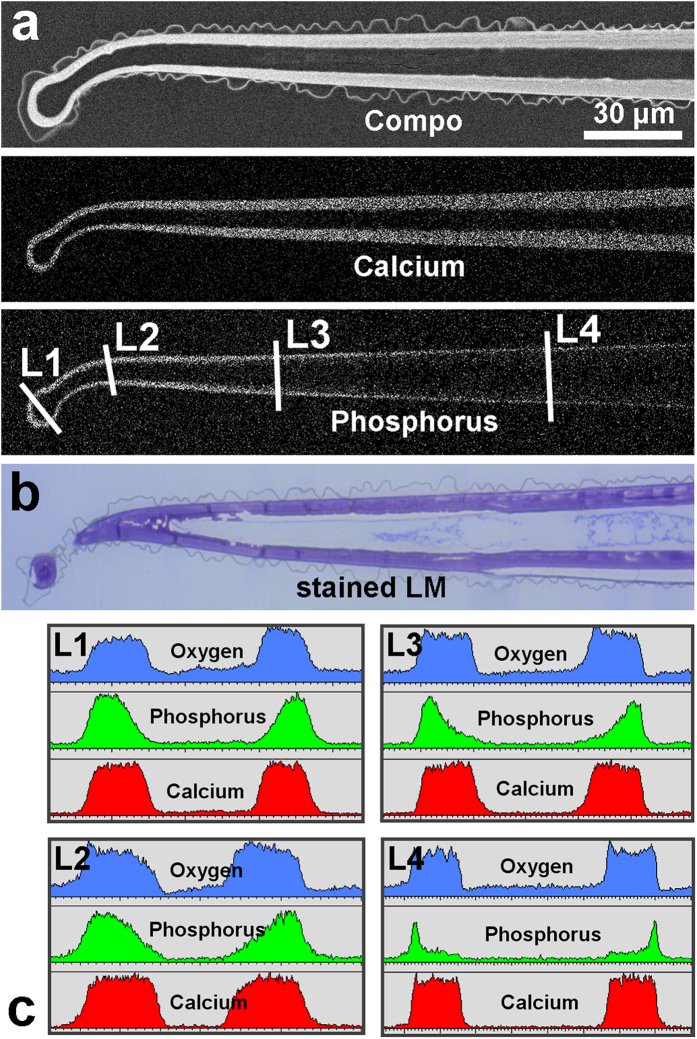
Analyses of a median longitudinal section through an embedded *Loasa pallida* stinging hair. (**a**) The SEM image of the sectioned block face shows the high mineral content in the wall by the compositional contrast of the BSE image. The element mapping images show the Ca and P distribution. (**b**) The light microscopy image of a toluidine blue-stained thin section (not exactly in the median plane) indicates that the entire cell wall contains organic material in addition to the mineral components. (**c**) EDX line scans show the concentration profiles for Ca, P, and O at different positions (L1 to L4). Note the continuous decrease of the phosphorus concentration from the outside towards the inner wall (line scan L3).

Scanning electron microscope (SEM) images of the surfaces of several *Loasa* species show a dense coverage with highly diversified trichomes such as small glochids and much larger stinging hairs (Fig. 1). Dual-detector SEM images, color-coded based on the back-scattered electron (BSE) intensity, display areas in yellow and red colors that contain higher concentrations of heavier elements such as calcium and phosphorus. In fact, SEM-based EDX micro-analyses revealed that all trichomes except the multicellular glandular trichomes contain high concentrations of calcium and phosphorus, particularly in the apex of the stinging hair and in the hooks of the glochidiate trichomes, but no silicon which would indicate the occurrence of silica. The shafts of both the glochidiate and the stinging hairs contain predominantly calcium, oxygen and carbon, but only very little phosphorus and again no silicon. High-resolution element distribution images from the glochidiate trichomes further show that phosphorus is restricted to the sharp, retrorse barbs on the glochidiate trichomes, whereas calcium is present along the entire cell wall (Fig. 2). The distribution of these elements in the cell wall is visualized in more detail in a longitudinal section through an embedded stinging hair (Fig. 3). The homogeneously bright appearance of the main cell wall in the BSE image indicates a high phosphorus and/or calcium content. Indeed, the calcium distribution image shows a uniform concentration across the entire cell wall, whereas a high phosphorus concentration was found at the apex and in the bulbous appendage. With increasing distance from the tip, most of the phosphorus is concentrated in the outer layer of the wall. The additional content of organic material is indicated by toluidine-blue staining of a thin section prepared for light microscopy (Fig. 3b).

**Figure 4:**
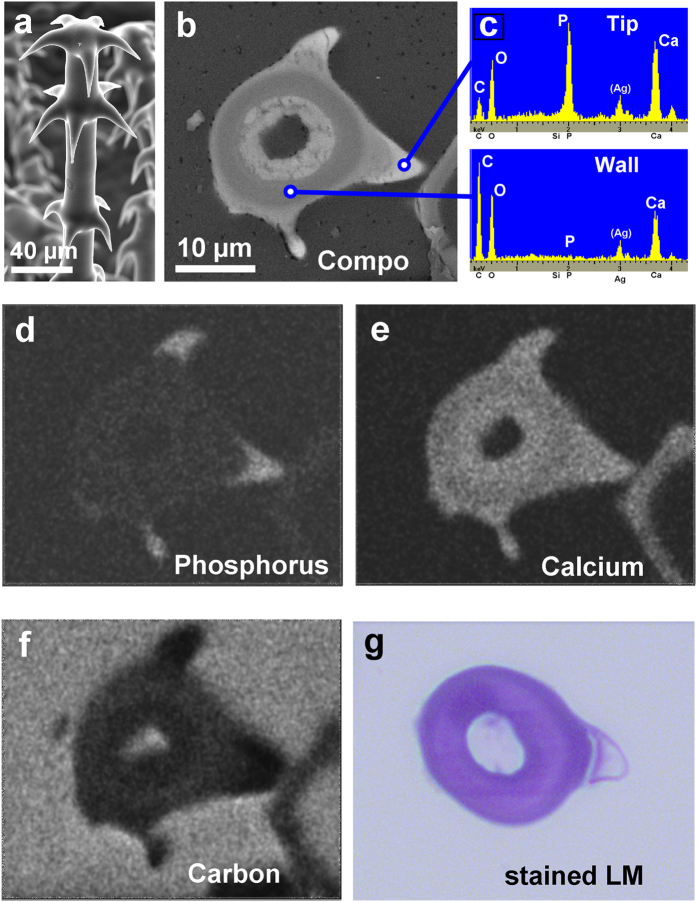
Analyses of a section through an embedded glochidiate trichome with hooks. (**a**) Surface view of a glochidic trichome. (**b**) BSE image of the sectioned block face showing compositional contrast. Higher brightness indicates higher content of the mineral elements Ca and P in the hooks. (**c**) EDX spectra of the hook tips and the main wall show high calcium phosphate and low carbon concentration in the hook tips. (**d**–**f**) Element mapping images show high P concentration and low C concentration in the hook tips. The Ca concentration is high in the hooks and slightly lower in the main wall. (**g**) The toluidine blue-stained thin section of another glochid indicates organic material in the main wall, whereas the hook tip appears almost unstained and free of carbohydrates.

EDX line scans across the shaft at different positions along the trichome revealed a relative homogeneous distribution of calcium and oxygen within the cell walls, whereas the phosphorus concentration smoothly decreases from distal to proximal and from the outside of the cell walls towards the lumen (Line scans L1 to L4 in Fig. 3). Similarly, BSE and element distribution images of a cross section of an embedded glochidiate trichome show the highest concentration of heavier elements (Ca, P) in the tips of the hooks (Fig. 4b–d), but very low carbon concentration. Accordingly, the stained thin section for light microscopy (Fig. 4g) shows organic material (carbohydrates) in the main wall, whereas the hook tip appears unstained and thus almost free of organic material. It follows that both the tips and hooks of the trichomes consist of highly concentrated calcium phosphate.

A comparison of EDX spectra of stinging hair tips and glochidiate trichome hooks of *Loasa* with those from the most important calcium phosphate minerals reveals that they have a Ca(Kα)-to-P(Kα) X-ray intensity ratio very similar to that obtained from apatite and tooth enamel (Supplementary Fig. 2), suggesting the occurrence of an apatite-like calcium phosphate phase. All *Loasa* species examined, including additional herbarium samples from South America, displayed the same chemical patterns as here demonstrated for *L. pallida*. In contrast, other genera of Loasaceae species such as *Caiophora* contain calcium and phosphorus in the stinging hairs, but silicon in the small trichomes (Supplementary Fig. 3), which is in accordance with the report of silica in Loasaceae trichomes by Thurston and Lersten^14^.

**Figure 5:**
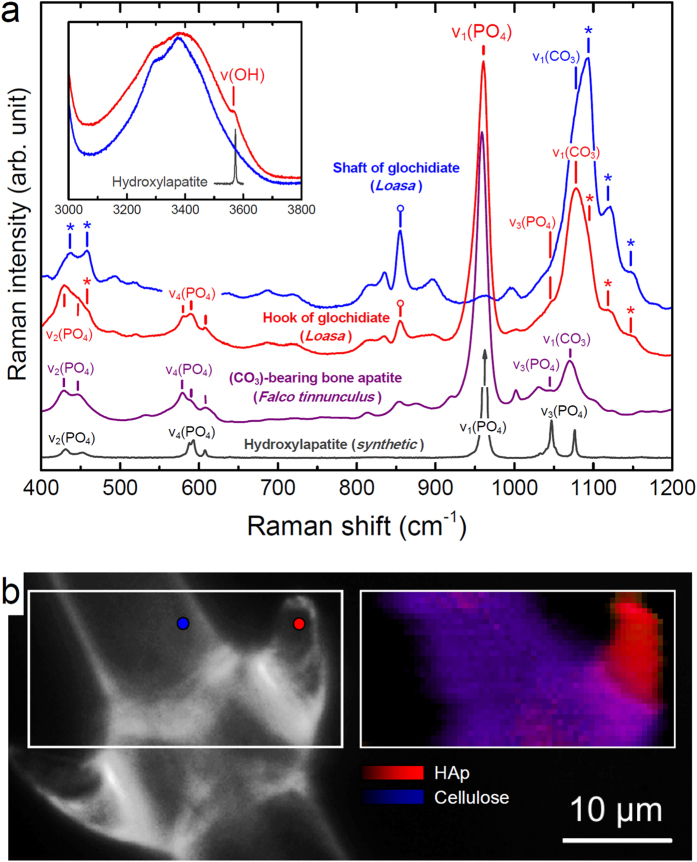
Raman spectra from *Loasa* trichomes in comparison with reference substances. (**a**) Representative Raman spectra from a hook and the shaft of glochidiate trichomes (*Loasa*) and from synthetic hydroxylapatite (HAp) and carbonated bone apatite. Location of spectra is shown in (**b**). Spectra were normalized with respect to the strongest band in the spectral range. Note the perfect agreement of the observed frequency and width of the internal [PO_4_] bands, including the intense symmetric ν_1_(PO_4_) stretching band near 958 cm^−1^, in the spectrum of the hook with those in the spectrum of carbonated bone apatite (=959 cm^−1^), which is clear evidence for nanocrystalline apatite in the trichome. The visible spectral differences are mainly related to the different organic environment. In bone apatite the Raman bands are partly convoluted with bands from collagen, whereas in the spectra from glochidiate trichomes the apatite bands are partly overlain by bands from cellulose and pectin, some of which are marked in the figure by (*) and (o), respectively. Note the occurrence of a band in the OH region in the spectrum from the hook (inset diagram) near 3566 cm^−1^ that is located on the top of the O-H stretching bands of cellulose. Although this band is much broader than the OH band of synthetic HAp near 3573 cm^−1^, it is only visible in spectra showing the strong ν_1_(PO_4_) band near 958 cm^−1^. It thus further identifies the apatite as hydroxylated apatite. Additionally, a band near 1078 cm^−1^ that is overlain be the strong cellulose band near 1093 cm^−1^ is detected. This band is likely related to the symmetric ν_1_(CO_3_) stretching mode of the carbonate molecule. (**b**) Optical image of hooks on a glochidiate trichome of *Loasa* (left) and corresponding false-color hyperspectral Raman image (right), showing the concentration of nanocrystalline hydroxylated apatite inside the hooks. The image was color-coded according to the integrated intensity of the v_1_(PO_4_) band and the sum of the integrated intensity of the cellulose bands near 1090, 1120, and 1155 cm^−1^. Raman spectra shown in (**a**) where taken from locations marked by colored circles in the optical image.

To unambiguously identify the calcium phosphate phase and to obtain information on its short- and long-range structural order, the trichomes were also studied by confocal Raman spectroscopy, X-ray diffraction (XRD), and transmission electron microscopy (TEM). Raman spectra of the hooks of glochidiate trichomes (Fig. 5a) and the tips of the stinging trichomes (Supplementary Fig. 4) show almost all characteristic internal (PO_4_) bands of hydroxylapatite (HAp), Ca_10_(PO4)_6_(OH)_2_, including an intense fully symmetric stretching ν_1_(PO_4_) band near 958 ± 1 cm^−1^ (962 cm^−1^ in HAp) as well as an OH band near 3566 ± 1 cm^−1^ (3573 cm^−1^ in HAp; Supplementary Table 1). In addition, the Raman spectra from the hooks and tips are virtually indistinguishable from spectra of bone apatite (Fig. 5a) which is known to be nano-crystalline with crystallite sizes between 2 to 200 nm^8^. The visible spectral differences are mainly related to the different organic environment (cellulose *versus* collagen). Compared with crystalline HAp, the Raman spectra of the hooks of glochidiate trichomes are characterized by considerably broadened and frequency-shifted Raman bands, including the OH band and the ν_1_(PO_4_) band that is asymmetric towards the low frequency side (Supplementary Table 1). Broadened and frequency-shifted Raman bands are characteristic for disordered, nanocrystalline materials, but also for amorphous materials^24^. The observed frequency of the ν_1_(PO_4_) mode of 959 ± 0.5 cm^−1^ unambiguously verifies the crystalline nature of the calcium phosphate, as in amorphous calcium phosphate (ACP) the ν_1_(PO_4_) mode vibrates with a significantly lower average frequency of 951 cm^−1^ (Supplementary Table 1)^25^. Moreover, based on a calibration of the Raman frequency of the ν_1_(PO_4_) band against the HAp crystallite size^25^, a crystallite dimension smaller than about 20 nm can be estimated.

False-color hyperspectral Raman images reveal the almost exclusive occurrence of HAp in the hooks (Fig. 5b), which is in agreement with the measured high phosphorus (and calcium) concentration. These observations are clear evidence that the phosphorus distribution in the trichomes directly reflects the quantity of nanocrystalline hydroxylated apatite. It is further noted that an intense ν_1_(CO_3_) carbonate band can be detected near 1072 cm^−1^ in the Raman spectra from the hooks of the trichomes, which, however, is superimposed by the intense C-C and C-O stretching bands of cellulose (Fig. 5a; Supplementary Table 1). In comparison with bone apatite, this suggests, at the first glance, that (CO_3_)^2−^ groups are also incorporated in the plant apatite. However, the decoupling of the phosphorus and calcium concentration across the cell walls (Fig. 3) rather implies the occurrence of a distinct amorphous carbonate phase together with nanocrystalline hydroxylated apatite. The Raman spectra of the stinging hair tips are very similar to those from the hooks, but with less contributions from organic material (Extended data Fig. 4). Further away from the tip, however, Raman spectra are characterized by an even broader and further red-shifted ν_1_(PO_4_) band (ν_1_ = 955 ± 1 cm^−1^) along with a more intense and broad ν_1_(CO_3_) carbonate band (Supplementary Table 1). It is noteworthy that the spectra strikingly resemble Raman spectra from ACP-based composites in a crayfish mandible^26^, suggesting the occurrence of both amorphous calcium phosphate and amorphous carbonate. In contrast, Raman spectra from the trichome shafts exhibit no apatite band, but a strong broad ν_1_(CO_3_) carbonate band as well as bands from cellulose. This in agreement with the Ca and P distribution measured by EDX and clearly demonstrates that in the shafts amorphous carbonate is the only inorganic phase.

**Figure 6:**
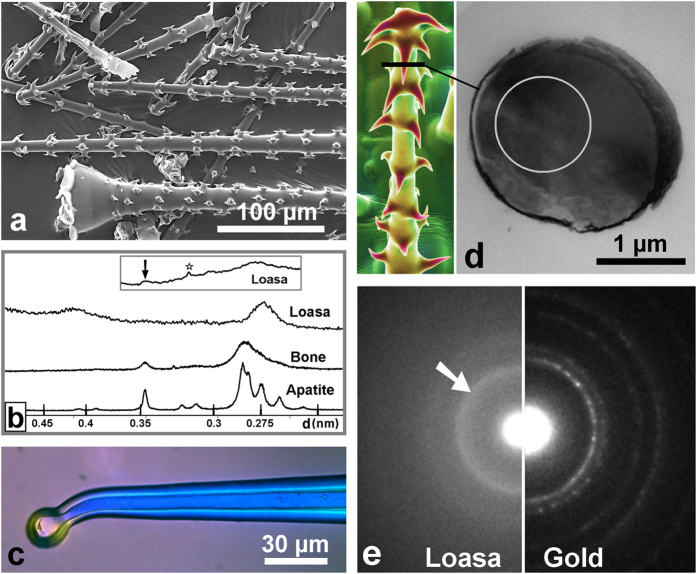
Results of ultrastructural examinations of *Loasa pallida* trichomes. (**a**) A typical batch of isolated glochidiate trichomes and stinging hair tips, which were used for X-ray diffraction. (**b**) Powder X-ray diffraction diagram from *Loasa* trichomes compared with that of synthetic apatite and bone apatite of cattle. The broad peak near *d* = 0.27 nm in the *Loasa* spectrum indicates nanocrystalline apatite, whereas the smaller broad peak near *d* = 0.42 nm is characteristic for ordered cellulose. Note that stinging hair tips normally yielded a similar spectrum. The detection of the (002) peak of the apatite structure in Loasa trichomes required a long-term measurement over 24 h. The diagram in the inset shows a low broadened peak around 0.34 nm (arrow). Another sharp peak at *d* = 0.314 nm (asterisk) resulted from sample contamination with silicon debris. (**c**) A stinging hair under a polarization microscope. The shiny blue color is characteristic for ordered cellulose in the cell wall. (**d**) Thin sections of a hook of a glochidiate trichome were used for selected area electron diffraction (SAED). (**e**) SAED pattern of a hook (left) compared with a SAED pattern from gold (right), showing a diffraction ring (arrow) that indicates randomly oriented nano-crystallites. The ring diameter reflects a lattice spacing, *d*, of 0.27 nm, which is in accordance with the XRD results (Fig. 6b). The innermost ring in the gold pattern reflects a *d* value of 0.236 nm.

Powder X-ray diffraction patterns of a batch of isolated glochids (Fig. 6a,b) as well as stinging hair tips typically showed only two broad diffraction peaks. The first peak corresponds to a lattice spacing, *d*, of about 0.42 nm, which matches with the average *d* value known for cellulose^27^. The crystalline nature of the cellulose is also reflected by the blue interference colors of the cell walls of the stinging hair shafts observed under the polarization microscope with crossed polarization (Fig. 6c). The second broad peak has its maximum at about 0.275 nm. This *d* value is in the range of the *d* values of the most intense diffraction peaks of HAp between 0.272 and 0.281 nm, but is significantly smaller than the *d* value measured on a bone sample as well as the *d* value reported for ACP of about 0.293 nm (30.5° 2Θ, Cu-Kα_1_ radiation)^28^. The reason for this shift is not yet resolved; it may reflect a lesser degree of order in certain crystallographic directions due to the interaction with organic components. Modeling of the X-ray diffraction pattern of nanocrystalline HAp by the Rietveld technique revealed a single broad diffraction peak with a maximum at a *d* value of ~0.279 nm (~32° 2Θ, Cu-Kα_1_ radiation) only for HAp crystallites with dimensions clearly smaller than about 5 nm^29^, corresponding to less than about six unit cells. Diffraction peaks from such small crystals, however, are significantly broader than the observed diffraction peak from the isolated glochids. The detection of the characteristic (002) peak of the apatite structure required a long-term measurement with 120 s counting time per 2θ step. The result is shown in the inset in Fig. 6b. The (002) peak with a *d* value of 0.346 nm now appears as a broad hump, as expected for nanocrystalline, hydroxylated apatite. Moreover, selected area electron diffraction (SAED) analyses by transmission electron microscopy (TEM) of hooks from a glochidiate trichome (Fig. 6d) revealed a broad diffraction ring (arrow in Fig. 6e), indicating randomly oriented nano-crystallites. Note that the diameter of the electron diffraction ring corresponds to a *d* value of 0.275 nm, which agrees well with the value obtained from X-ray diffraction data (Fig. 6b).

Whatever the exact dimensions and habit of the nanocrystallites, both the Raman spectroscopic and diffraction data clearly prove the occurrence of nanocrystalline calcium phosphate with a hydroxylapatite-like structure. In addition, the occurrence of additional amorphous calcium phosphate phases or composites is indicated. The data further suggest that the here identified plant apatite nano-crystallites are more disordered and/or smaller (<20 nm) than those in most bones which generally yield XRD patterns that are characterized by clear resolvable diffraction peaks (Fig. 6b)^30^.

## Discussion

Element composition, the vibrational Raman spectrum, and X-ray and electron diffraction patterns evidently demonstrate, for the first time, the presence of a nanocrystalline apatite-cellulose composite material in the hooks of glochidiate trichomes as well as in the tips of the stinging hairs of Loasaceae. In contrast to sporadic occurrence of diffuse traces of phosphorus in other plant trichomes, nanocrystalline hydroxylated apatite appears to play a central functional role as a structural biomineral in Loasaceae. Five of the six species of *Loasa* so far examined exhibit a similar mineralization pattern that is characterized by complete absence of silica, but a high concentration of calcium phosphate in all stinging hairs and trichome tips. All species of the related genera *Caiophora* and *Blumenbachia* so far examined have calcium phosphate stinging hair tips, but silica in the hooks of the small trichomes, sometimes together with calcium phosphate.

There is little doubt about the primarily anti-herbivore function of Loasaceae trichomes. It is striking, however, that they appear to be optimized for this function not only in morphology, but also in the detailed patterns of biomineralization. The shaft of the stinging hairs is impregnated with calcium carbonate and possibly other calcium compounds, forming a stiff and inelastic container for the liquid. The apex of the stinging hair needs to be both hard and brittle, and only in this apical region calcium carbonate is partly or fully replaced by nanocrystalline apatite. Similarly, the sharp hooks on the glochidiate trichomes are made of the hardest available biominerals, either hydroxylated apatite for *Loasa* or silica in the case of *Caiophora*, indicating a strict genetic control of mineralization.

The trichome cell walls of Loasaceae provide very useful systems for studying the processes of biomineralization, since they are highly accessible for observations during mineral deposition. The distinct localization of nanocrystalline calcium phosphate in the trichomes and the divergent chemical composition in closely related plant species also opens up new ways to study the process of trichome biomineralization. The species-dependent differences in the formation of either silica or calcium phosphate in homologous structures may even be the starting point for studies on the genetic control of biomineralization. The interplay of calcium phosphate and carbonate seems to be important for the formation and differential physical properties of these structures, such as the unusually small size of the apatite crystallites. Several publications deal with stabilization of amorphous calcium carbonate by phosphate compounds^31^,^32^ or with the use of calcium carbonate as a seed for the growth initialization of calcium phosphate^33^, e.g., for the growth of artificial bone substitutes. The observations here documented provide a natural model system for these investigations. Biomimetic composite materials based on calcium phosphate with some type of organic scaffolding for use as, e.g., bone substitutes, are currently being intensively investigated^34^,^35^,^36^,^37^,^38^,^39^,^40^,^41^,^42^,^43^,^44^. Calcium phosphate composites based on cellulose appear to be a useful alternative to protein-based materials due to their likely higher biocompatibility. Recently, attempts have been made to specifically use cellulose scaffolding for HAp mineralization^45^. However, until now a natural model system for this was not known. The natural cellulose-calcium phosphate composite material documented here provides such a model system for the first time. The valuable properties of biological calcium phosphate materials, such as ivory or tooth enamel, suggest a considerable potential of these composites for the development of advanced biomimetic materials for a range of different purposes and as possible alternatives to organic polymers. Accordingly, the report of structural biomineralization with nanocrystalline hydroxylated apatite in Loasaceae trichomes opens new avenues for a whole range of investigations, including biomimetic material and functional traits of plant surfaces and their genetic control.

## Materials and Methods

### Plant material

All plants presented here were cultivated at the Botanische Gärten der Universität Bonn: *Loasa heterophylla* (Accession 35601 - 8 – 2012; *Loasa pallida* (Accession 36565 - 2 – 2011); *Caiophora coronata* (Accession 36587 - 2 – 2011.); *Urtica dioica* (Accession 4919 - 2 – 1989.), all species are vouchered at the herbarium of the Nees-Institut für Biodiversität der Pflanzen (BONN).

### Sample preparation

Particular attention was paid for a reliable preparation of samples in order to avoid artifacts. The stinging hairs are a chemically instable system. Highly reactive CaCO_3_ in the cell wall is separated from the acidic vacuole liquid only by a thin cytoplasma layer. Any damage of the cell can thus cause chemical reactions with precipitations of insoluble calcium compounds. Therefore, calcium phosphate is detected in damaged or dead stinging hairs of plants that have a high phosphate content in the cell lumen, such as the common stinging nettle (*Urtica dioica*); (see Supplementary Fig. 1f). While EDX element distribution mapping required stable dried samples, we confirmed the results by spot measurements on fresh hydrated and frozen, hydrated samples, precluding artifacts. The analysis of ultrathin sections with a TEM required ‘dry sectioning’, because otherwise thin sections floating on water may lose their minerals.

### Scanning electron microscopy

Scanning electron microscopic imaging and EDX analyses were performed with a Cambridge S 200 or a LEO 1450 scanning electron microscope each equipped with a SE, a BSE, and an EDX detector (Oxford Instruments, UK) and with Link ISIS software. Investigations were carried out either on fresh hydrated leaves that withstand drying inside the SEM for more than 30 minutes or on frozen hydrated samples at approximately −100 °C in a custom-made cryo stage. The detailed procedures are published elsewhere^46^. Element mapping by EDX required samples with better long-term stability. Therefore, stinging hairs and trichomes were isolated. Individual stinging hairs were cut off and washed immediately to remove most of the vacuole liquid. The small trichomes were harvested from deeply-frozen leaves by scraping them off the leaf lamina with a knife blade. The samples were usually sputter-coated with silver or palladium, because, in contrast to gold, the EDX spectra of these metals do not interfere with the characteristic X-ray peaks of certain interesting elements such as silicon and phosphorus. Fresh and frozen hydrated samples were used without metal coating or with Ag or Pd coating of less than 15 nm, which is sufficiently transparent for back-scattered electrons necessary for BSE imaging.

### Raman spectroscopy

Raman spectra from different parts of the trichomes, synthetic hydroxylapatite, and carbonated bone hydroxylapatite of a kestrel (*F. tinnunculus*) (courtesy of A. Schmitz, Institute for Zoology, University of Bonn) were collected with a confocal Horiba Scientific HR800 Raman spectrometer at the Steinmann Institute of the University of Bonn, Germany. The Raman effect was excited with a 2 W frequency-doubled solid state Nd:YAG laser (532.09 nm), whereby the laser power was adjusted to less than 20 mW at the sample surface. The scattered Raman light was detected by an electron multiplier charged-coupled device (EM-CCD) after being dispersed by a grating with 600 grooves/mm and having passed a 100 μm spectrometer entrance slit. With these parameters the spectral resolution was 3.5 cm^−1^, as empirically determined by the full width at half maximum of the neon lines. The spectrometer was calibrated using the first order Si band at 520.7 cm^−1^. A 100× objective with a numerical aperture of 0.9 was used for all measurements, resulting in a diffraction-limited lateral resolution in the order of 0.8 μm. Hyperspectral Raman imaging was carried out with a step size of 0.5 μm and counting times of two times 0.5 s per pixel with maximal amplification of the EM-CCD.

### Powder diffraction analysis

Powder diffraction diagrams of isolated trichomes were first recorded with a PW 1049/10 diffractometer with Bragg-Brentano geometry at the Institute for Inorganic Chemistry, University of Bonn, Germany, using Co-Kα radiation (wavelength 1.79 Å). The diffraction diagrams were recorded with steps of 2*θ* = 0.02° and acquisition times between 1 and 10 s per step. A second long-term measurement was carried out with a Siemens D5000 powder diffractometer with a graphite secondary monochromator at the Steinmann Institute of the University of Bonn, Germany, using Cu-Kα radiation (wavelength 1.54 Å). The trichomes were spread on a Si wafer and the diffraction pattern was recorded between 24 and 38° 2*θ* with an aquisition time of 120 s per 2*θ* step, which was necessary to detect the (002) peak of hydroxylapatite.

### Transmission electron microscopy

For TEM electron diffraction analyses and a general examination of cross sections of isolated stinging hairs and trichomes, the trichomes were washed in water, dehydrated with acetone, and embedded in an ‘AGAR Low Viscosity Kit’ (Plano GmbH, Wetzlar, Germany). Dry sectioning was performed with a diamond knife with an ultramicrotome. TEM analyses of ultra-thin sections were then carried out with a Philips CM 300 at the Institute for Inorganic Chemistry, University of Bonn, Germany, to study the crystalline structure by selected area electron diffraction (SAED) measurements.

## Additional Information

**How to cite this article**: Ensikat, H.-J. *et al.* A first report of hydroxylated apatite as structural biomineral in Loasaceae – plants’ teeth against herbivores. *Sci. Rep.* **6**, 26073; doi: 10.1038/srep26073 (2016).

## Supplementary Material

Supplementary Information
