# Supplementary material for: A first report of hydroxylated apatite as structural biomineral in Loasaceae – plants’ teeth against herbivores
Source: Sci Rep. 2016 May 19;6:26073. doi: 10.1038/srep26073 (PMC4872142; doi:10.1038/srep26073)
Supplement: Supplementary Information [file srep26073-s1.pdf]

Supplementary Information

Title:

A first report of hydroxylated apatite as structural biomineral in Loasaceae – plants´ teeth against herbivores

Authors:

Hans-Jürgen Ensikat, Thorsten Geisler, and Maximilian Weigend

Supplementary Figure 1: *Urtica dioica* stinging hairs with phosphate traces and calcium phosphate precipitations.

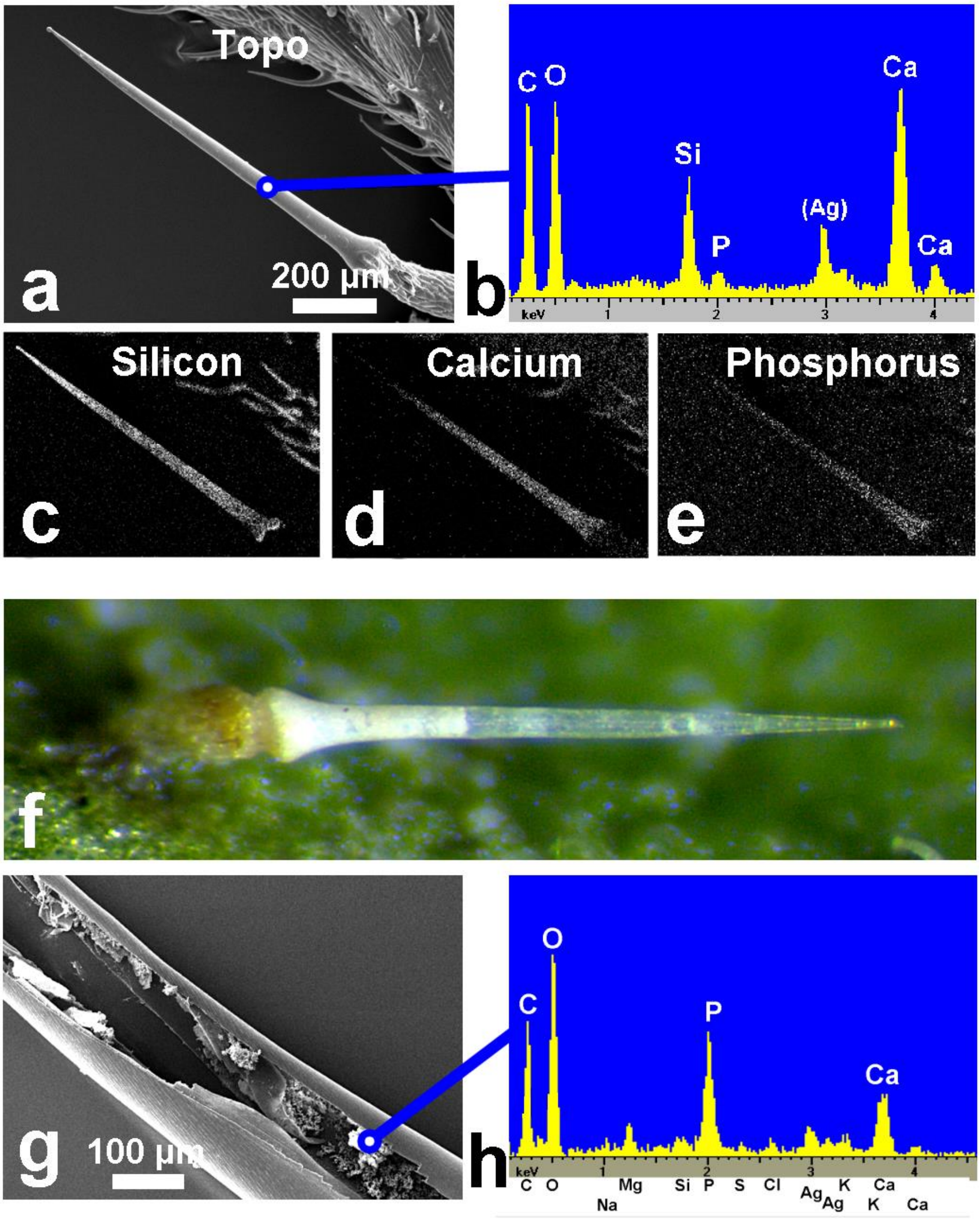

(a-e) EDX analyses and element mapping images show a minor phosphate concentration together with calcium in some stinging hair walls. (f-h) White precipitations in dead stinging hairs can be seen under a stereo microscope. The EDX spectrum of the material on the inside of a dead stinging hair indicates concentrated calcium phosphate. Thus calcium phosphate can occur in stinging nettles, but it was not found to build functional structures.

Supplementary Figure 2: Comparison of the P-to-Ca ratio in the hooks of *Loasa* glochidiate trichomes versus other calcium phosphate minerals.

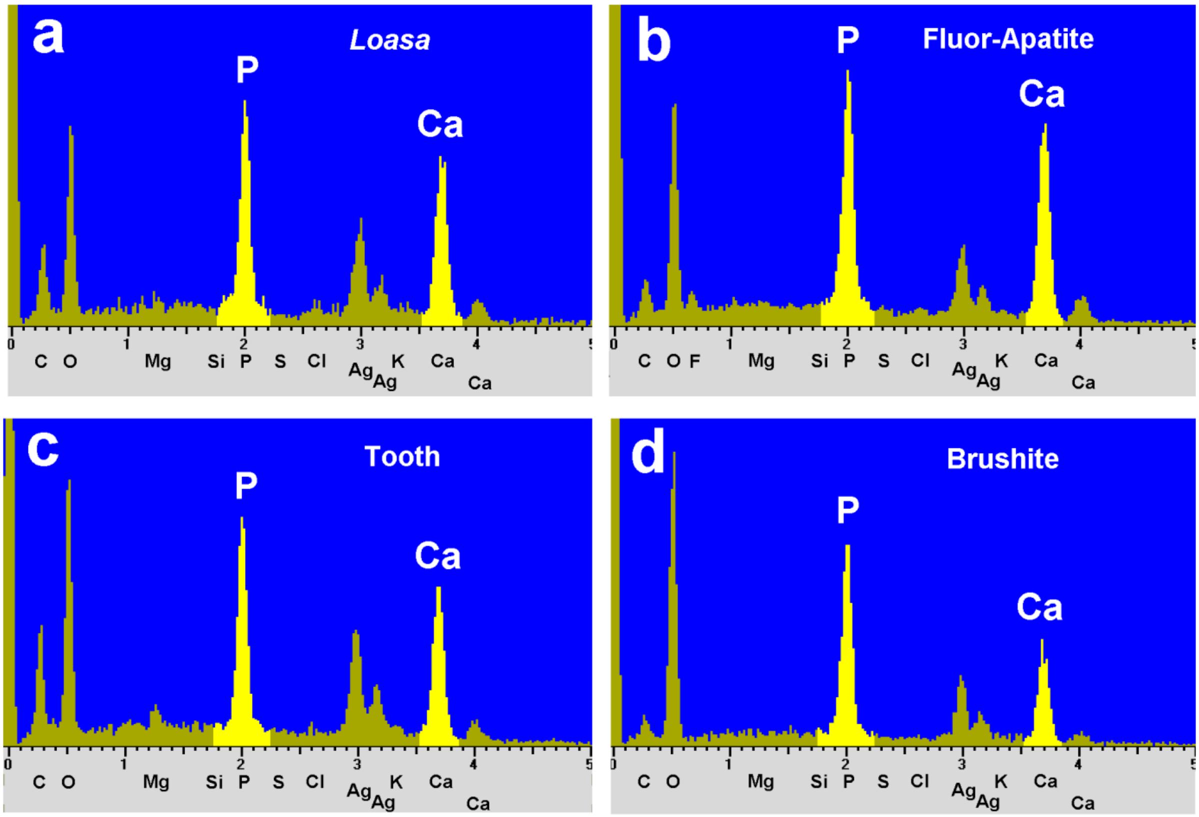

(a) *Loasa heterophylla* hook tips, (b) natural fluor-apatite $Ca_5(PO_4)_3F$, (c) enamel of a human tooth, (d) synthetic brushite $CaHPO_4 \cdot 2H_2O$. All spectra measured with electron energies of 10 keV. Note that the P(Kα)-to-Ca(Kα) peak height ratio of the P-richest spots of *Loasa* trichomes is similar to that of apatite, but does not reach that of brushite.

Supplementary Figure 3: Trichomes of *Caiophora coronata* (Loasaceae) leaves containing both, calcium phosphate and silica.

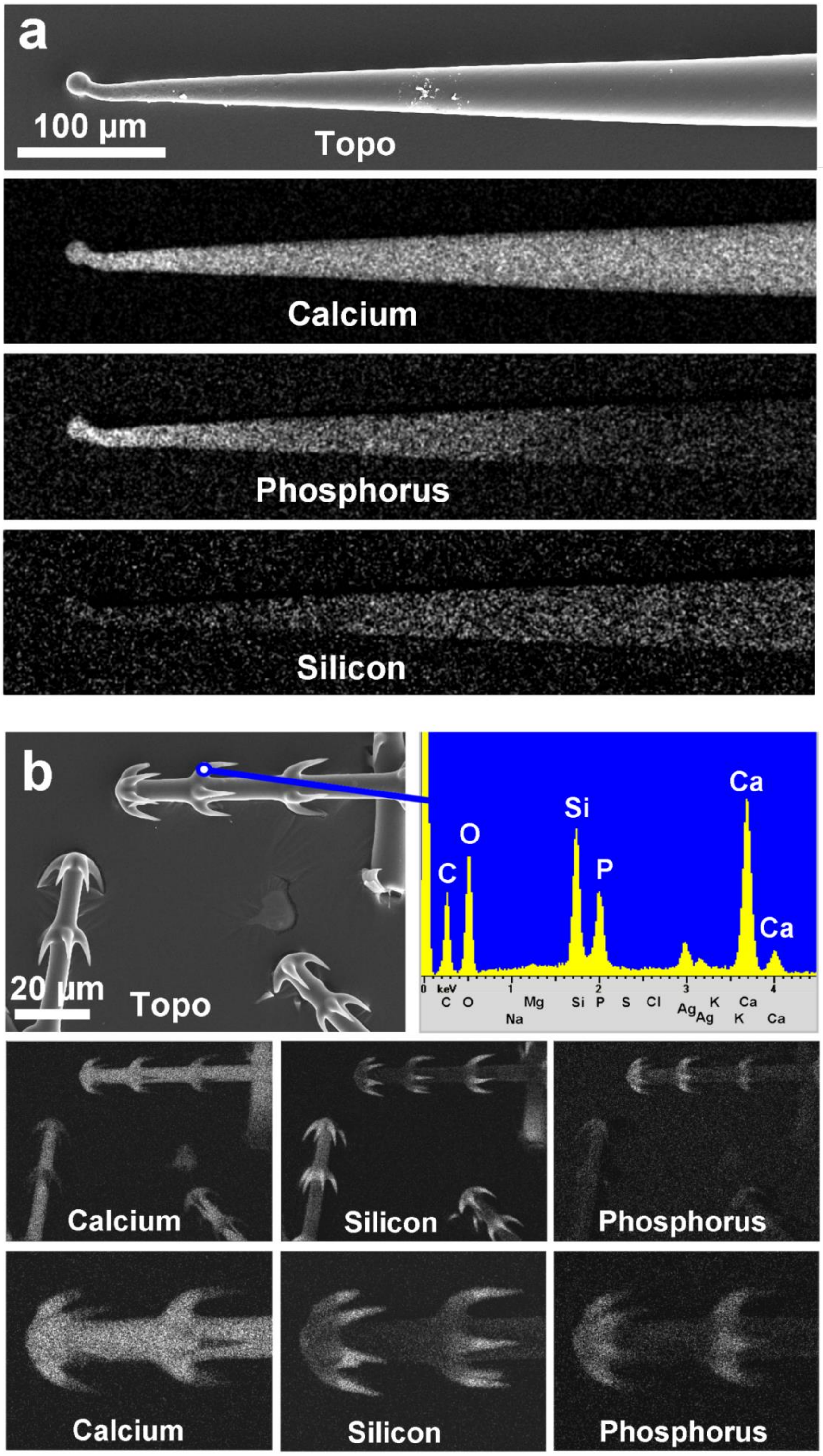

(a) The apical region and tips of stinging hairs containing calcium phosphate, low concentrations of silica are detected in the shaft. (b) The glochidiate trichomes contain calcium carbonate in the shafts and silica in the tips of the hooks. Some trichomes contain both, silica in the tips, and calcium phosphate in the basal part of the hooks.

Supplementary Figure 4: Representative Raman spectrum from a tip and shaft of a stinging hair in comparison with a spectrum from synthetic hydroxylapatite (HAp) and a carbonated bone apatite.

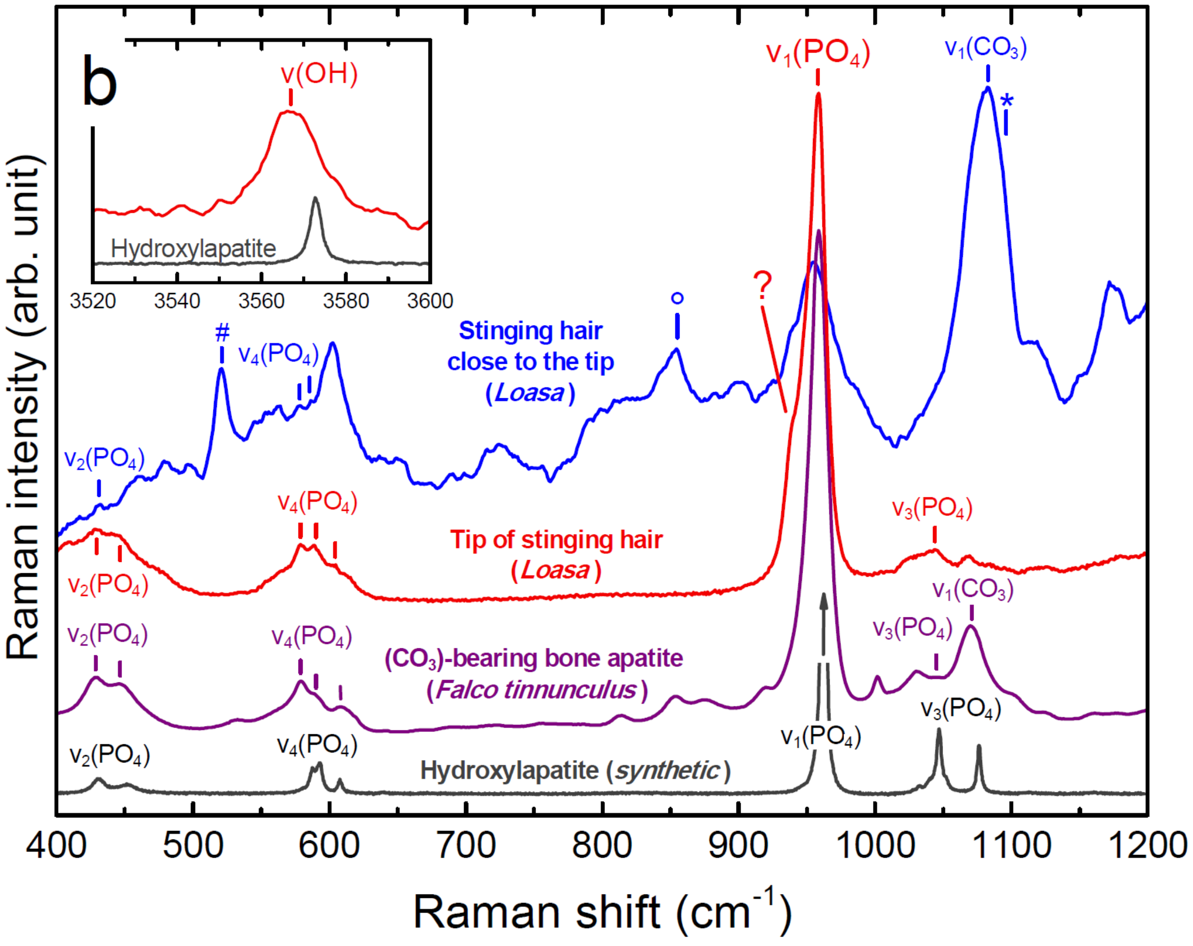

Spectra were normalized with respect to the strongest band in the spectral range. Note that in the spectrum from the stinging hair the HAp bands are partly overlain by bands from cellulose, pectin, and lignin, some of which are marked by (*), (o), and (#), respectively, and the occurrence of a still unidentified band (shoulder) near 940 cm^{-1}.

Supplementary Table 1: List of observed Raman band frequencies from different parts of the trichomes compared with Raman bands from a synthetic hydroxylapatite (HAp), a carbonated bone HAp of a kestrel (*F. tinnunculus*), and amorphous calcium phosphate (ACP). Observed characteristic fingerprint band frequencies for organic compounds are also listed.

Synthetic HAp	Carbonated bone HAp	ACP[1]	Hook of glochidiate	Tip of stinging hair	Stinging hair close to tip	Assignment
			(cm^{-1})			
430	428	429	429	429	431	$v_2(PO_4)$
452	446		447	446		
587	579	573	580	579	578	
593	590		590	589	586	$v_4(PO_4)$
608	608		608	603		
				940	940	?
962	959	951	958	958	955	$v_1(PO_4)$
1032						
1041	1045	1050	1045	1044		
1047						$v_3(PO_4)$
1052						
1076				1069		
3573			3566	3567		v(OH)
	1070		1078		1082	$v_1(CO_3)$
			855		855	Pectin
			1094		1093	Cellulose
					1605	Lignin

[1] Data from Kazanci et al. 2006 (25)
